# New Partners Identified by Mass Spectrometry Assay Reveal Functions of NCAM2 in Neural Cytoskeleton Organization

**DOI:** 10.3390/ijms22147404

**Published:** 2021-07-09

**Authors:** Antoni Parcerisas, Alba Ortega-Gascó, Marc Hernaiz-Llorens, Maria Antonia Odena, Fausto Ulloa, Eliandre de Oliveira, Miquel Bosch, Lluís Pujadas, Eduardo Soriano

**Affiliations:** 1 Department of Cell Biology, Physiology and Immunology, University of Barcelona and Institute of Neurosciences, 08028 Barcelona, Spain; albaortega@ub.edu (A.O.-G.); marchernaiz@gmail.com (M.H.-L.); fausto.ulloa@ub.edu (F.U.); lluis.pujadas@ub.edu (L.P.); 2 Centro de Investigación Biomédica en Red sobre Enfermedades Neurodegenerativas (CIBERNED), 28031 Madrid, Spain; 3 Department of Basic Sciences, Universitat Internacional de Catalunya, 08195 Sant Cugat del Vallès, Spain; miquelbosch@uic.es; 4 Plataforma de Proteòmica, Parc Científic de Barcelona (PCB), 08028 Barcelona, Spain; maodena@pcb.ub.es (M.A.O.); eoliveira@pcb.ub.es (E.d.O.)

**Keywords:** NCAM2, mass spectrometry, cytoskeleton, neuronal morphogenesis, MAP2, CaMKIIα, Neurofilaments, Nogo

## Abstract

Neuronal cell adhesion molecule 2 (NCAM2) is a membrane protein with an important role in the morphological development of neurons. In the cortex and the hippocampus, NCAM2 is essential for proper neuronal differentiation, dendritic and axonal outgrowth and synapse formation. However, little is known about NCAM2 functional mechanisms and its interactive partners during brain development. Here we used mass spectrometry to study the molecular interactome of NCAM2 in the second postnatal week of the mouse cerebral cortex. We found that NCAM2 interacts with >100 proteins involved in numerous processes, including neuronal morphogenesis and synaptogenesis. We validated the most relevant interactors, including Neurofilaments (NEFs), Microtubule-associated protein 2 (MAP2), Calcium/calmodulin kinase II alpha (CaMKIIα), Actin and Nogo. An in silico analysis of the cytosolic tail of the NCAM2.1 isoform revealed specific phosphorylation site motifs with a putative affinity for some of these interactors. Our results expand the knowledge of NCAM2 interactome and confirm the key role of NCAM2 in cytoskeleton organization, neuronal morphogenesis and synaptogenesis. These findings are of interest in explaining the phenotypes observed in different pathologies with alterations in the NCAM2 gene.

## 1. Introduction

Neuronal differentiation, and the establishment of cell polarity and synaptic connections, are crucial events for the development of the brain [1,2]. These processes are tightly regulated by numerous factors, including cytoskeleton proteins, membrane receptors and elements of the extracellular matrix. Cell Adhesion Molecules (CAMs) are a family of membrane proteins with many functions that transduce extracellular and membrane-bound signals into cellular events, such as membrane remodeling, cytoskeletal rearrangement and dynamics, vesicular transport, gene expression and cell survival [3,4,5].

The immunoglobulin superfamily of cell adhesion molecules (IgSF CAM) includes more than 50 different members in mammals [6]. IgSF CAMs are characterized by an extracellular region with one or several immunoglobulin-like (Ig) domains, followed by fibronectin type III (Fn3) domains. Most IgSF CAMs present a single transmembrane domain with an intracellular tail, while other members are anchored to the cell membrane through a glycosylphosphatidylinositol (GPI) anchor [7]. Typically, the extracellular and the intracellular domains of IgSF CAMs interact with several proteins, ligands or modifiers, which determine their important roles during development [5,8,9].

The Neural Cell Adhesion Molecule (NCAM) family has two members, NCAM1 and NCAM2 [10,11]. Both proteins present a similar extracellular structure with five Ig domains and two Fn3 domains. NCAM1 has three different isoforms (180, 140 and 120 KDa), whereas NCAM2 has two isoforms: NCAM2.1 (with a transmembrane domain and a cytoplasmic tail) and NCAM2.2 (a shorter isoform with no transmembrane domain but a GPI-anchor motif) [12,13].

A large number of studies have investigated the functions of NCAM1, which play a fundamental role in both neural development and plasticity [8,14,15]. NCAM1 interacts with many extracellular ligands and adaptors, such as the Fibroblast Growth Factor Receptor (FGFR), Prion Protein (PRNP), Homer1, Tyrosine-protein kinase Fyn or Focal adhesion kinase 1 (FAK) [16,17,18,19,20]. By contrast, NCAM2 is less studied. NCAM2 is widely expressed in the Central Nervous System (CNS) during brain development. In the olfactory system NCAM2 is necessary for the formation and maintenance of dendritic and axonal compartments [21,22,23,24,25,26], and in synapse formation and maintenance [26,27,28]. Genetic studies suggest that the NCAM2 gene is implicated in the intellectual disability phenotype in Down syndrome and Autism Spectrum Disorders, as well as in other neurodevelopmental diseases [24,29,30,31,32]. In addition, NCAM2 has been involved in synaptic deficits in Alzheimer’s disease [28]. Although NCAM2 functions have begun to be clarified [25,26,28], the extracellular ligands and intracellular adaptors that interact with NCAM2 remain largely unknown. It has been recently shown that NCAM2 regulates neurite outgrowth through the kinase Src in the cerebral cortex [26,27], and that it mediates dendritic morphogenesis via the formation of molecular complexes with Microtubule-associated protein 2 (MAP2) and 14-3-3 family proteins [25].

To gain insight into NCAM2 functions, here we investigate the interactome of the NCAM2 protein during postnatal cortical development using proteomic and molecular approaches. We found more than 100 proteins that interact with NCAM2 using mass spectrometry. We further validated the more relevant interactions for cytoskeleton organization (CaMKIIα, NEFs, Actin and MAP2) by means of immunoprecipitation. To better characterize the NCAM2 interactome, our proteomic data were analyzed by bioinformatic tools; we detected significant enrichments in gene ontology terms and in cellular pathways linked to the cytoskeleton, as well as to other important neural functions. In addition, we identify putative phosphorylation sites of the NCAM2.1 cytosolic tail using in silico analysis. These data increase our knowledge about the interactome of the NCAM2 protein and open new perspectives for the study of NCAM2 functions.

## 2. Results

### 2.1. In Silico Analysis of the Cytoplasmic Domains of NCAM2.1

**Figure 1:**
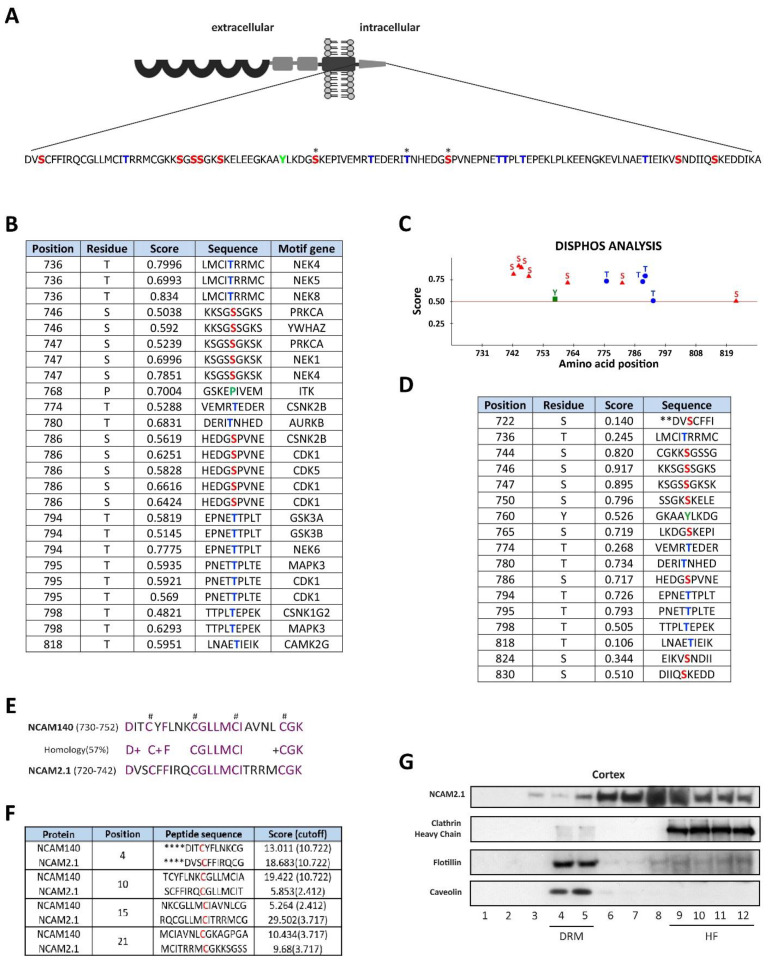
In silico analysis of NCAM2.1 cytoplasmatic domain. (**A**) Schematic representation of phosphorylation sites in NCAM2.1 cytoplasmatic tail amino acid sequences. Serine (red), Tyrosine (green) and Threonine (blue) phosposites are represented. * phosphorylated sites previously described. (**B**) In silico analysis using Scansite 4.0 to identify motifs gene interactors (motif gene) that are likely to be phosphorylated by specific protein kinases or binding domains such as SH2 domains, 14-3-3 domains or PDZ. (**C**,**D**) Schematic representation and Table of phosphorylation sites of NCAM2 cytoplasmatic tail with their in silico predicted scores using Disphos. (**E**) Comparison of NCAM140 (730–752 amino acids) and NCAM2.1 (719–741 amino acids) sequences, NCAM1 has an intracellular region with palmitoylation modification sites that contain cysteine-residues which are critical for its localization in lipid rafts and also present in NCAM2.1 cytoplasmatic domain (#). (**F**) In silico analysis of NCAM2.1 and NCAM1 with their predicted scores using CSS-Palm software 4.0. NCAM2.1 contains the same potential palmitoylation sites similarly to NCAM1 with high scores (**G**) WB of NCAM2.1 from cortical extracts subjected to sucrose gradient. NCAM2.1 co-signals in lipid rafts (lanes 4–5, DRM, Detergent-resistant membrane, identified by Flotillin and Caveolin), but mainly outside lipids rafts (lanes 6–12, HF, High Fraction).

Previous studies showed the importance of the cytoplasmic domains of CAMs, such as NCAM1 (NCAM140 or NCAM180 isoforms). We thus performed a structural analysis of the amino acid sequence of the NCAM2.1 cytosolic domain (Figure 1A). The residues S765, T780 and S786 have already been described as phosphorylation sites [33]. We used different bioinformatic tools (Scansite 4.0 and Disphos 1.3 analysis, Koch Institute and MIT, Cambridge, MA, USA) to identify additional phosphorylatable residues. High scores for S746 and T818, which match the consensus sequences of 14-3-3ζ and CaMKIIγ, respectively, were detected with Scansite 4.0. Disphos analysis showed that approximately 70% (12/17) of the phosphorylatable residues have significant scores to be phosphorylated (serines 7/9, threonines 4/7 and tyrosine 1/1), as shown in Figure 1B–D. We also detected different motifs that match the consensus sequences of the kinases Cyclin dependent kinase 5 (CDK5), Protein kinase C alpha (PRKCA), Casein kinase II (CSNK2) and Glycogen synthase 3 (GSK3), using Scansite. These are kinases known to phosphorylate other CAMs, such as Cadherins, Epithelial cell adhesion molecule (EpCAM), Integrins or Neuronglia cell adhesion molecule (NgCAM) [34,35,36,37,38,39].

CAMs are known to have different roles depending on their membrane localization in lipids rafts [40,41,42,43], as in the case of NCAM140. Since the NCAM2 molecule is a paralog of NCAM1, we decided to compare the intracellular domains of NCAM140 and NCAM2.1. Although the cytoplasmic domain sequences of both proteins share an homology of 54% (65/120 amino acids, data not shown), the phosphorylation sites are not found in the homologous regions. Therefore, we focused on the comparison of the NCAM1 sequences responsible for lipid raft localization, corresponding to 730–752 amino acids [41], with their homologous regions in NCAM2.1 (Figure 1E). NCAM1 has an intracellular region with palmitoylation modification sites that contains cysteine-residues that are critical for NCAM1 targeting to lipid rafts. We observed the presence of four similar cysteine residues in the homologous region of NCAM2.1, suggesting that these conserved residues could be implicated in lipid raft localization (Figure 1E). Using CSS-Palm 4.0 software (Sun Yat-sen University, Guangzhou, China), we detected with high scores the same palmitoylation sites in NCAM2.1 and NCAM1 (Figure 1F). In order to confirm the NCAM2.1 localization in lipid rafts, we analyzed the expression of NCAM2.1 by Western blot (WB) in cortical extracts subjected to sucrose gradient (Figure 1G). We found that NCAM2.1 co-localized with lipid raft markers (lanes 4–5), but was predominantly expressed outside lipids rafts (lines 6–12).

### 2.2. NCAM2 Interactome in Postnatal Cerebral Cortex

**Figure 2:**
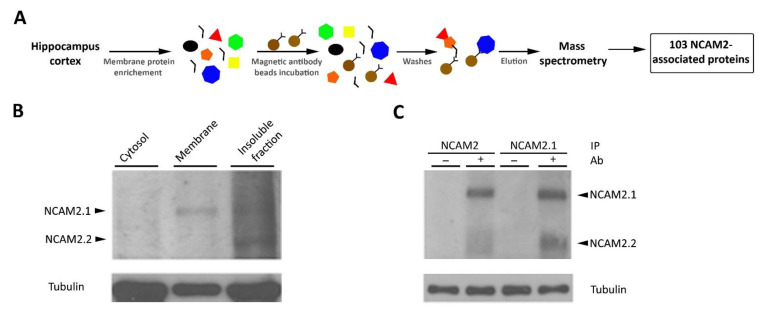
The interactome of NCAM2; the mass spectrometry approach. (**A**) Schematic representation of the mass spectrometry approach. Brain lysates from P10–12 mice were enriched in membrane proteins and incubated with magnetic beads, which were previously conjugated with an antibody against NCAM2.1 or against both NCAM2 isoforms. The eluted proteins were processed for mass spectrometry analysis. A list of 103 proteins that interact with NCAM2 was obtained with a False Discovery Rate ≤ 0.01%, Table 1. (**B**) WB detection of NCAM2.1 and NCAM2 proteins in the cytosolic, membrane and insoluble fractions of the brain lysates used for the mass spectrometry assay. NCAM2.1 isoform is detected in both fractions, the membrane and the insoluble fraction while NCAM2.2 is specially enriched in the insoluble fraction. Tubulin was used as a loading control. (**C**) WB detection of NCAM2 from samples of magnetic beads elution.

We performed protein immunoprecipitation and peptide detection as described [25] in Figure 2A. The membrane fraction was isolated from the cerebral cortex of postnatal P12–15 mice (Figure 2B) and NCAM2 was purified by immunoprecipitation with two different antibodies, one recognizing the cytoplasmic tail of NCAM2.1 (EB06991, Everest, Oxfordshire, UK), and the other recognizing the extracellular region of both NCAM2 isoforms (AF778, R&D Systems, Minneapolis, MN, USA). We used non-conjugated magnetic beads for control immunoprecipitations. Our data first confirmed the specific interaction of NCAM2.1 with NCAM2.2 as shown in Figure 2C. Immunoprecipitated proteins were then eluted and digested for mass spectrometry analysis. The resulting peptides were identified by LC-MS/MS to obtain putative protein partners (False Discovery Rate ≤ 0.01%) as represented in Figure 2A and Table S1.

We detected 103 proteins that specifically interact with NCAM2 (Table S1) and 52 of them were identified with two or more different peptides (Table 1). Many of the peptides corresponded to microtubule, intermediate filaments or Actin cytoskeletal proteins (i.e., Actin, ACTB; Tubulin beta-4A, TUBB4A; Alpha-internexin, INA; Tubulin alpha-1A, TUBA1A; Tubulin alpha-1C, TUBA1C;Neurofilament light polypeptide, NEFL; Neurofilament medium polypeptide, NEFM; and Tubulin beta-6 chain, TUBB6) and to cytoskeleton-associated proteins (i.e., Microtubule-associated protein 2, MAP2; Microtubule-associated protein 1B, MAP1B; F-actin-capping protein beta, CAPZB; and F-actin-capping protein alpha-2, CAPZA2). Interestingly, the analysis also detected the interaction with motor proteins (i.e., Dynein light chain 1, DYNLL1; Myosin light polypeptide 6, MYL6; and Dynein light chain 2, DYNLL2), kinases and adapter proteins (i.e., CaMKII family proteins and 14-3-3 family proteins), calcium-binding proteins (i.e., CaMKII family proteins, Calumenin, CALU; Reticulocalbin-2, RCN2; Calmodulin, CALM1; and Hippocalcin-like protein 1, HPCAL1) and transcription activity regulators (i.e., Elongation factor 1-beta, EEF1B; Nuclease-sensitive element-binding protein 1, YBX1; Elongation factor 1-delta, EEF1D; and Eukaryotic translation initiation factor 3 H, EIF3H).

A total number of 16 proteins were detected with both NCAM2 antibodies, and 20 proteins were detected in both independent experiments (Table 1 and Table S2). Moreover, seven out of these 20 proteins were also detected with both antibodies (ACTB, TUBA1A, HSPA8, HSBP1, GRN and DYNLL1). Specifically, Actin (ACTB), Heat shock cognate 71 kDa protein (HSPA8) and Granulin (GRN) proteins were identified in all the replicates and with all antibodies, confirming the strong reliability of these detected interactions (Table 1 and Table S2). Additionally, mass spectrometry analysis showed different protein coverage among the detected NCAM2 interactors (Table 1 and Table S2).

### 2.3. NCAM2 Interacts with Cytoskeleton and Cytoskeleton-Associated Proteins

**Figure 3:**
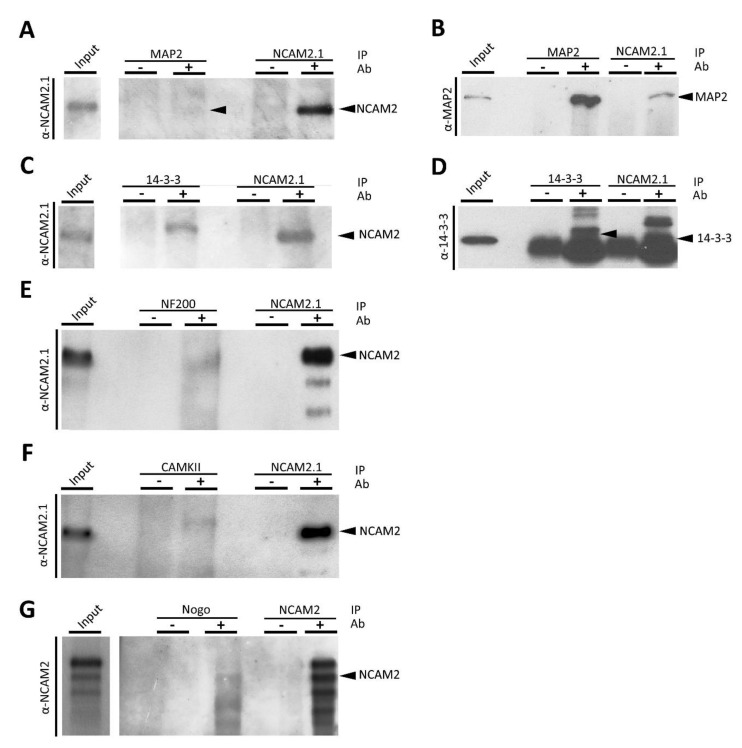
NCAM2 interacts with MAP2, 14-3-3, NFs, CaMKII and Nogo. (**A**) WB detection of NCAM2.1 in different co-immunoprecipitation experiments with MAP2 (**A**), 14-3-3 (**C**), NEFs (**E**), CaMKII (**F**) or NEFs (**G**). Detection of MAP2 (**B**) or 14-3-3 (**D**) in different co-immunoprecipitation experiments with NCAM2.1 from P10–15 mouse cortex and hippocampal protein extracts. Immunoprecipitation using MAP2 (**A**), 14-3-3 (**C**), NEFs (**E**) or CaMKII (**F**) or Nogo (**G**) antibodies confirmed the presence of NCAM2.1 protein in the WBs after detection with NCAM2.1 or NCAM2 antibodies (**G**). Immunoprecipitation using NCAM2.1 antibodies confirmed the presence of MAP2 (**B**) and 14-3-3 (**D**).

Mass spectrometry analysis identified some proteins with high robustness. These proteins are MAP2, Neurofilaments, and CaMKII. MAP2 is the protein with the largest number of peptides detected in the present analysis. We already described the interaction of MAP2 with NCAM2 (Figure 3A,B), as well as the interaction of NCAM2 with 14-3-3 family proteins (Figure 3C,D).

We previously reported that NCAM2 regulates microtubule polymerization and stability [25]. The present data show that NCAM2 additionally interacts with different key cytoskeletal components. The cytoskeleton proteins ACTB, TUBA1A and ACTC1 were detected in different replicates. We confirmed the NCAM2-Actin interaction by immunoprecipitation, IP, using specific antibodies for NCAM2 (Figure S1). Besides the interaction with Actin filaments, our results detected the interaction of NCAM2 with CAPZA and CAPZB, Actin-interacting proteins forming a heterodimer that binds to the barbed-ends of Actin filaments, which block their polymerization and depolymerization [44,45]. CAPZ proteins play a relevant role in growth cone dynamics, dendritic spine development and synapse formation [46,47].

A large number of peptides corresponding to NEFL and NEFM were identified in the different experiments. Neurofilaments are important for the radial growth and the stability of axons and enable effective and high-velocity nerve conduction [48,49]. The NCAM2-NEFs interactions were validated by IP and WB (Figure 3E).

The CaMKII family of proteins was also identified with different peptides. CaMKIIα and CaMKIIβ play crucial roles in neuronal morphogenesis and plasticity [1,50,51]. Previous results showed the functional relation between NCAM2 and CaMKII, important for neurite branching and filopodia formation [26]. Here, our results show a direct interaction between these proteins, validated by IP and WB as shown in Figure 3F.

We detected Reticulon 4 (Nogo) with more than one peptide when the NCAM2.1 isoform was immunoprecipitated. The Nogo-NCAM2.1 interaction was additionally assessed by immunoprecipitation of Nogo and NCAM2 detection by WB (Figure 3G). Nogo proteins are important for neuron migration and neurite outgrowth and branching [52].

Finally, Heat shock cognate 71 kDa protein and Granulin were detected in all conditions. HSPA8 is a member of the chaperon family and participates together with HSC70 in protein folding and degradation, stress response and chaperone-mediated autophagy [53,54]. GRN is a secreted growth factor involved in different neurological functions.

### 2.4. Bioinformatic Analysis of the NCAM2 Interactome

A bioinformatic analysis was performed with the 52 proteins detected with two or more peptides using String 11.0 (Elixir, Hinxton, Cambridgeshire, UK). [55].

#### 2.4.1. Bit Map

**Figure 4:**
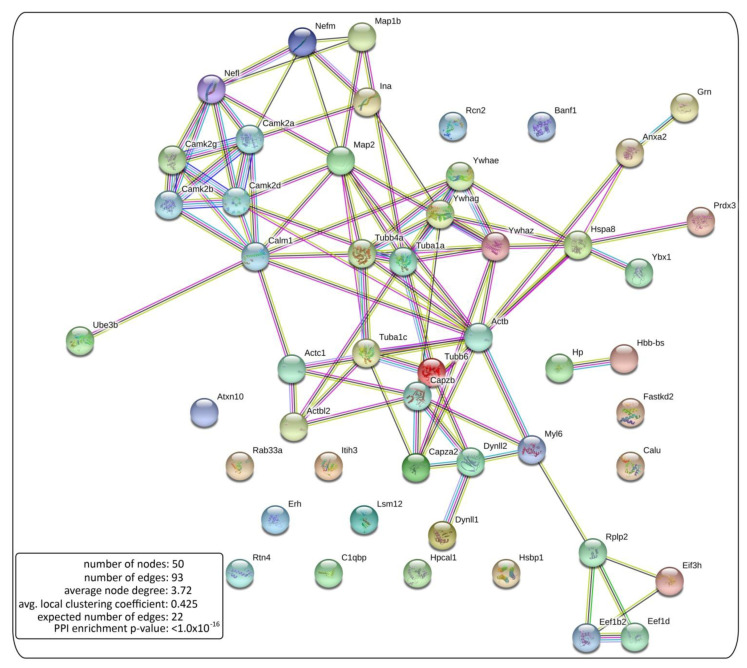
NCAM2 protein interaction network. Bitmap representation of the NCAM2 interaction network. The network edges mean the evidence of interactions between the proteins of Table 1, proteins detected with two or more peptides in the mass spectrometry assay. Our bitmap shows a significant increased number of edges, 93 edges with our pull versus 22 expected in random condition.

To visualize the proteomic results, we obtained a high quality graphic of NCAM2 interactions in bitmap format, showing a significant increased number of edges: 93 edges with our pull versus 22 expected in random condition (Figure 4). This significant increase shows that NCAM2 interacts with different protein complex previously described.

#### 2.4.2. Gene Ontology Terms

We next performed Gene Ontology (GO) and pathway enrichment analyses. We found a significant enrichment for Biological Process (Table 2 and Table S3); Molecular Function (Table 2 and Table S4); and Cellular Components GO terms (Table 2 and Table S5). Focusing on Biological Process GO terms, our results show that the partners of NCAM2 are significantly enriched in 118 biological process terms (Table 2 and Table S3), including cell differentiation and neuronal morphogenesis. Moreover, NCAM2 interactors are significantly enriched in GO terms linked to cytoskeleton, corroborating the above findings. We also found terms related to the organization and localization of plasma membrane proteins.

Regarding Molecular Function, presented in Table 2 and Table S4, NCAM2-related proteins are associated with cytoskeleton functions including structural constituents of cytoskeleton (GO:0005200), cytoskeletal protein binding (GO:0008092), cytoskeletal regulatory protein binding (GO:0005519), scaffold protein binding (GO:0097110) and Actin binding (GO:0003779), as well as with translation processes: translation elongation factor activity (GO:0003746), translation factor activity and RNA binding (GO:0008135GO:0003723).

Furthermore, NCAM2 protein interactors are enriched in 78 significant cellular components, as shown in Table 2 and Table S5. Again, NCAM2 partners are classified as cytoskeleton cellular components, cellular components involved in neuronal morphogenesis and maintenance, and synaptogenesis and synaptic maintenance processes.

#### 2.4.3. Pathways Analysis

The results of the Kyoto Encyclopedia of Genes and Genomes (KEGGS) pathways enrichment analysis (Table 3 and Table S6), and the Reactome pathway enrichment analysis (Table 3 and Table S7) are also displayed. Our data show an enrichment in 71 different pathways, including pathways related to calcium and neurotransmitter receptors, cell cycle regulation and to Rho GTPases (Table 3 and Table S7).

Taken together, our results suggest that NCAM2 plays a significant role in the organization of the cytoskeleton and it is involved in calcium signaling and membrane dynamics. These processes are essential for the proper neuronal morphogenesis, synapse formation and neuronal network maintenance.

## 3. Discussion

In the present study, we explored the interactome of NCAM2 and revealed a significant role of this protein in the organization and dynamics of the cytoskeleton. The mass spectrometry approach showed that NCAM2 interacts with key cytoskeleton components and with a large number of other intracellular proteins. These observations point to NCAM2 functions as crucial for different cytoskeleton-related functions, including neuronal differentiation and maintenance, and synaptogenesis. In addition, our data suggest that NCAM2 may act as a putative receptor for GRN. Our results reveal that NCAM2 interacts with more than 100 proteins in murine cortical samples at two weeks of postnatal development. Since 56% of the identified proteins with two or more peptides were also detected in different experimental conditions, we believe that the method employed here is robust and the resulting data consistent. Nonetheless, it is important to mention that the extraction protocol used in this assay led to an enrichment of the NCAM2.1 isoform compared with NCAM2.2. This is due to the fact that NCAM2.1 is present in the soluble fractions, while NCAM2.2 is more frequently found in lipid rafts (the insoluble fraction), which makes it more difficult to purify.

The results obtained in this work also contribute to a better characterization of NCAM2 protein functions. As previously described, NCAM2.1 bears a transmembrane domain and an intracellular cue, while NCAM2.2 lacks the transmembrane domain and binds to the membrane by a GPI anchor [21,23]. Those differences are important for the localization and possible functions of the isoforms. Our study suggests that the transmembrane isoform, NCAM2.1, could also be found in lipid rafts. The in silico analysis of the amino acid sequence of NCAM2.1 shows similarities in some cysteine residues with NCAM140. The cysteine residues adjacent to the transmembrane domain in NCAM140 are palmitoylation modification sites and are important for targeting the protein to lipid rafts [41,42,56]. Lipid raft localization is crucial for cellular signaling and for the interactions of cell adhesion molecules with other ligands, which, in turn, activate different pathways [41]. The localization of NCAM2.1 in lipid rafts, suggested by the analysis of the amino acidic sequence, was confirmed by the detection of this isoform in lipid rafts. Moreover, the in silico analysis of the NCAM2.1 sequence shows a high percentage of putative residues that are likely to be phosphorylated, some of them coincident with previously described phospho-sites, which could be relevant to better understand NCAM2 functions [33].

Recent studies have determined the importance of NCAM2 in neuronal polarization, cell morphogenesis and in the formation and maintenance of excitatory glutamatergic synapses [25,27,28]. NCAM2 has been reported to induce local calcium spikes through the activation of Src, and to regulate microtubule stability through the formation of a protein complex with MAP2 and 14-3-3 [25]. Here, we reveal the interaction of NCAM2 with a number of other proteins, including cytoskeleton components and cytoskeleton-associated proteins, kinases, translation factors, growth factors and other intracellular components. Regarding the interactions with the cytoskeleton, our data indicate that NCAM2 interacts with Actin and NEFs. The interactions of NCAM2 with Actin and NF200 were validated using immunoprecipitation. Consistent with these results, a reduction of NCAM2-altered Actin cytoskeleton produced an aberrant growth cone mobility in NCAM2-deficient neurons [25]. NCAM2 also interacts with proteins that modulate cytoskeleton function and dynamics, or motor proteins, including MAP2, MAP1B, CAPZA, CAPZB, DYNLL1 and DYNLL2. These proteins are necessary for processes, such as neuronal survival, growth cone extension, axon elongation, autophagy or synapses maintenance. The bioinformatic analysis confirmed the strong relationship between NCAM2 and the cytoskeleton by showing a significant enrichment in terms and pathways related to cytoskeleton organization and dynamics [57,58,59,60,61].

Alterations in cytoskeleton dynamics are found in some pathologies, such as Autism Spectrum Disorders (ASD) or other neurodevelopmental diseases, including lissencephaly [62,63,64]. For example, the dynamics of Actin polarization is impaired in cells from ASD patients [62]. Consistent with these observations, genetic analyses showed that deletions and single nucleotide polymorphism in NCAM2 gene are detected in ASD patients. Our results suggest that alterations of NCAM2 may cause destabilization or modification of the cytoskeleton, thereby affecting neurodevelopment.

In this study, we detected several NCAM2-interacting proteins that turn out to be linked to synaptogenesis and synaptic plasticity processes. The most relevant ones are CaMKII, HSPA8, MAP1B, CAPZ and elongation factors, such as EEF1B, EEF1D, EIF3H and EIF3F. NCAM2 is highly expressed in the adult brain and highly localized to synapses [28,65]. We detected a strong interaction of NCAM2 with CaMKII in our proteomic analysis. Previous studies have shown that NCAM2 can activate CaMKII [26]. CaMKII is a well-known regulator of neuronal differentiation, synaptogenesis and synaptic plasticity [66].

We detected an interaction of NCAM2 with HSPA8. HSPA8 is a member of the chaperons’ family and participates together with HSC70 in protein folding and degradation. HSC70 accumulates in presynaptic buttons and catalyzes the release of Clathrin from Clathrin-coated synaptic vesicles, an event that is necessary for the synaptic vesicle-recycling pathway [67,68]. Other key cytoskeleton proteins found to interact with NCAM2 are MAP1B and CAPZ. Both have an important role in synaptogenesis and synaptic plasticity [46,69]. CAPZ is a capping protein that stabilizes Actin fibers [70] located in the postsynaptic density [71]. Neuronal activity induces its accumulation in the spines, facilitating the remodeling of these postsynaptic structures [72]. The loss of a subunit from the CAPZ complex led to alterations in dendritic spine formation and to defects in the specification of the presynaptic and postsynaptic structures [73].

14-3-3 proteins are involved in the regulation of Cofilin phosphorylation and the stabilization of Actin filaments. The knock-out of 14-3-3 protein in murine models results in a reduction of the dendritic tree complexity and the number of spines. In addition, animals deficient in 14-3-3 proteins bear behavior problems linked to major susceptibility to develop schizophrenia [74,75,76].

Another novel finding from our mass spectrometry assay is the interaction of NCAM2 with local protein synthesis components, which is a crucial mechanism for synaptic plasticity and other neuronal functions [77]. We detected different proteins involved in translation, such as EEF1B, EEF1D, EIF3H and EIF3F. Overall, our data suggest that NCAM2 plays a key function in the process of synaptogenesis and in the maintenance of synapses and plasticity in adult stages. Amyloid-beta increases NCAM2 cleavage and reduces its function in synapses, which could explain the synaptic loss observed in early stages of Alzheimer’s disease [28].

Finally, GRN has been shown to participate in neurite outgrowth and branching, axon growth and synapses formation and maintenance. Furthermore, mutations in the GRN gene have been linked to frontotemporal dementia [78,79,80,81,82,83,84] and it has been proposed as a potential target for the treatment of those dementias. One of the proposed receptors for GRN is Sortilin 1 (SORT1) [85], but the induction of neurite outgrowth is not regulated by this receptor, which indicates that another receptor is involved in the process. Our proteomic results suggest that NCAM2 could act as a GRN receptor during neuronal differentiation.

In summary, the present study provides a detailed view of the interactome of NCAM2, and offers additional information about NCAM2 localization and structure. The observed interactions of NCAM2 with cytoskeleton proteins, growth factors and other intracellular components increase our knowledge about this cell adhesion molecule and contribute to explain its functions during brain development and synaptic plasticity. More analyses and studies will be necessary in order to understand the complexity of the NCAM2 interactome in other developmental periods or brain regions. The present work adds substantially to our understanding of NCAM2 and lays the groundwork for a better characterization of NCAM2 functions in the central nervous system during development and adult stages, as well as the implications of this protein in neuronal diseases.

## 4. Materials and Methods

All experimental procedures were carried out following the guidelines of the Committee for the Care of Research Animals of the University of Barcelona, in accordance with the directive of the Council of the European Community (2010/63 y 86/609/EEC) on animal experimentation. The experimental protocol was approved by the local University Committee (CEEA-UB, Comitè Ètic d’Experimentació Animal de la Universitat de Barcelona) and by the Catalan Government (Generalitat de Catalunya, Departament de Territori i Sostenibilitat).

### 4.1. Antibodies

The following commercial primary antibodies were: anti-14-3-3 (1657, SantaCruz, Dallas, TX, USA); anti-Actin (MAB1501, Chemicon International-Fischer Scientific, Waltham, MA, USA); anti-CaMKIIα (M1-048, ThermoFisher Scientific, Waltham, MA, USA); anti-Caveolin (ab2910, Abcam, Cambridge, UK); anti-Clathrin (610500, BD Biosciences, Franklin Lakes, NJ, USA), anti-Flotillin (610820, BD Biosciences, Franklin Lakes, NJ, USA), anti-MAP2 (M9942 clone HM-2, Sigma-Aldrich, Sant Louis, MO, USA); anti-NCAM2 (AF778, R&D Systems, Minneapolis, MN, USA)); anti-NCAM2.1 (EB06991, Everest, Oxfordshire, UK); anti-NF200 (N4142, Sigma-Aldrich, Sant Louis, MO, USA) and anti-Nogo (11027, Santa Cruz, Dallas, TX, USA).

### 4.2. Mass Spectrometry Assay

Protein immunoprecipitation and analysis were performed as described [25]. Briefly, hippocampus and cortex regions were dissected and homogenized in an isotonic buffer (Tris 10 mM a pH 7,4, KCl 10 mM, MgCl2 1.5 mM, EGTA 1 mM) with protease inhibitors (Complete, Roche, Basel, Switzerland), using a Polytron. The supernatant with the cytosolic fraction was discarded and the membrane fraction was homogenized in a lysis buffer (Hepes 50 mM pH 7.5, 150 mM NaCl, 1.5 mM MgCl2, 1 mM EGTA, 10% glycerol, and 1% Triton X-100) in orbital agitation. Samples were centrifugated at 15,000 rpm for 15 min at 4 °C and the supernatant was selected.

For the mass spectrometry assay, magnetic beads (Dyneabeads Antibody Coupling Kit, Life Technologies, Carlsbad, CA, USA) were conjugated with an antibody against NCAM2 (AF778, R&D Systems, Minneapolis, MN, USA) or with an antibody against NCAM2.1 (EB06991, Everest, Oxfordshire, UK), according to the manufacturer’s instructions. The supernatant containing the membrane fraction previously obtained, was incubated with the conjugated magnetic beads o/n at 4 °C. After washing with the lysis buffer 3 times, proteins were eluted with 30 μL of a Urea buffer (urea 8 M, Tris 50 mM a pH 7.5, DTT 60 mM) during 15 min at room temperature. The samples were processed and analyzed at the Proteomic facility of PCB (Proteomics unit, Parc Cientific de Barcelona, Barcelona, Spain). Samples were digested with trypsin (2 μg, pH 8, 32.5 °C, o/n). The resulting peptides were separated by nanoUPLC (NanoAcquity, Waters, Milford, MA, USA) and detected with Orbitrap Velos (Thermo Fisher Scientific, Waltham, MA, USA). The detection was performed with a resolution of 60,000, a ratio 400 *m*/*z* and an acquisition of 300–1800 *m*/*z*. Protein lists were obtained with an FDR ≤ 0.01%. String 11.0 (Elixir, Hinxton, Cambridgeshire, UK) was used for the bioinformatics analysis. The most abundant detected protein was NCAM2 and it was not included in the list.

### 4.3. Immunoprecipitation for Western Blotting

The membrane fraction obtained as previously described, was incubated with 2 μg of the selected antibodies overnight (anti-NCAM2.1, anti-NCAM2, anti-MAP2, anti-14-3-3, anti-NF200, anti-CaMKIIα and anti-Actin). To precipitate the proteins, protein G-Sepharose beads (17-0618-01, GE Healthcare, Chicago, IL, USA) were added and samples were incubated for 2 h in orbital agitation. After washing with the lysis buffer, proteins were eluted with 20 μL of loading buffer (0.5 M Tris-HCl (pH 6.8), 2.15 M β-mercaptoethanol, 10% SDS, 30% glycerol, and 0.012% bromophenol blue) during 5 min at 95 °C and processed for Western blot. Samples were separated on 10% SDS-PAGE and transferred to nitrocellulose membranes (1620112; Bio-Rad, Hercules, CA, USA). Filters were blocked in a 5% dry milk-supplemented 0.1% Tween 20 PBS prior to immunoreaction and immunoblotted with antibodies against 14-3-3 (1:2000), Actin (1:5000), Map2 (1:1000), NCAM2 (1:500) and NCAM2.1 (1:1000). The membranes were incubated with HRP-labeled secondary antibodies (DAKO, Santa Clara, CA, USA) for 1 h at RT in TBST and developed with the ECL system (GE Healthcare, Chicago, IL, USA).

### 4.4. Sucrose Gradient for Lipid Raft Isolation

The cortex from CD1 mice were used for lipid raft isolation. Two cortices were homogenized in 3 mL of MES (2-morpholino ethanesulfonic acid)-buffered saline (34 mM, pH 6.5 and 0.15 mM NaCl) plus 1% Triton X-100 supplemented with Complete Protease Inhibitor Cocktail (11697498001; Basel, Switzerland). Sucrose was then added to achieve a final concentration of 40%. A 5–30% linear sucrose gradient was layered on top and centrifuged at 39,000 rpm for 16 h at 4 °C in a Beckman SwTi rotor. A total of 12 fractions, 1 mL each, were collected from the top and analyzed by Western blot, as previously described, using the following antibodies: NCAM2 (1:1000), Caveolin (1:5000), Flotillin (1:3000) and Clathrin (1:5000).

### 4.5. Bioinformatic Analysis

Genomes and proteomes were downloaded from the National Institutes of Health (NIH, Bethesda, MD, USA), Nation al Center for Biotechnology Information (NCBI, Bethesda, MD, USA) and UniProt database (Geneva, Switzerland). Proteins identified by two or more peptides obtained with FDR ≤0.01% were analyzed with String 11.0 [55]. The bit map was elaborated using basic settings. Network edges represent evidence interactions at medium confidence of the score (0.4). The whole genome of mice was assumed for the statistical background in the functional enrichment (Gene Ontology terms, Kyoto Encyclopedia of Genes and Genomes pathways and Reactome pathways). Genomes and proteomes were downloaded from the National Institutes of Health (NIH).

To identify NCAM2.1 phosphorylatable domains, the amino acid sequence of the NCAM2.1 cytoplasmatic domain (UniProtKB-O35136, NCAM2_MOUSE, 719–837 amino acids) was analyzed using ScanSite 4.0 at low stringency [86,87] and DISPHOS (DISorder-enhanced PHOSphorylation predictor) [88].

To compare the NCAM2.1 and NCAM140 cytoplasmic domains, amino acid sequences of NCAM2.1 (UniProtKB-O35136, NCAM2_MOUSE, 719–837 amino acids) and NCAM140 (UniProtKB-O35136, NCAM1_MOUSE, 730–810 and 1076–1115 amino acids) were analyzed with BLAST software (NIH) and CSS-Palm software 4.0.

### 4.6. Statistical Analysis

Statistical analysis was carried out using the GraphPad Prism 5 software (San Diego, CA, USA).

## Figures and Tables

**Table 1:** The interactome of NCAM2.

Protein (Gene)	Ptd	Function
Microtubule-associated protein 2 (Map2)	24	actin binding, calmodulin binding, cytoskeletal regulatory, microtubule binding and protein kinase binding
Neurofilament light polypeptide (Nefl)	20	structural constituent of cytoskeleton andstructural constituent of postsynaptic intermediate filament cytoskeleton
Actin, cytoplasmic 1 (Actb)	19	structural constituent of cytoskeleton and structural constituent of postsynaptic actin cytoskeleton
Neurofilament medium polypeptide (Nefm)	19	microtubule binding and structural constituent of cytoskeleton
Tubulin beta-4A (Tubb4a)	18	structural constituent of cytoskeleton and GTPase activity
Alpha-internexin (Ina)	17	structural constituent of cytoskeleton, structural constituent of postsynaptic actin and intermediate filament cytoskeleton
Tubulin alpha-1A (Tuba1a)	13	structural constituent of cytoskeleton
Tubulin alpha-1C (Tuba1c)	12	structural constituent of cytoskeleton
Actin, alpha cardiac muscle 1 (Actc1)	11	structural constituent of cytoskeleton and myosin binding
Calcium/calmodulin-dependent protein kinase type II beta (Camk2b)	11	calmodulin-dependent protein kinase activity, protein serine/threonine kinase activity and structural constituent of postsynaptic actin cytoskeleton
Calcium/calmodulin-dependent protein kinase type II alpha(Camk2a)	10	calmodulin-dependent protein kinase activity, glutamate receptor binding and protein serine/threonine kinase activity
Microtubule-associated protein 1B (Map1b)	9	actin binding, cytoskeletal regulatory protein and binding
Beta-actin-like protein 2 (Actbl2)	8	structural constituent of postsynaptic actin cytoskeleton
F-actin-capping protein beta (Capzb)	8	actin binding and beta-tubulin binding
14-3-3 protein zeta/delta (Ywhaz)	8	recognition of a phosphoserine or phosphothreonine motif
Tubulin beta-6 chain (Tubb6)	7	structural constituent of cytoskeleton
Calcium/calmodulin-dependent protein kinase type II delta (Camk2d)	6	calmodulin-dependent protein kinase activity and protein serine/threonine kinase activity
Calcium/calmodulin-dependent protein kinase type II gamma (Camk2g)	6	calmodulin-dependent protein kinase activity and protein serine/threonine kinase activity
Enhancer of rudimentary homolog (Erh)	5	Cell cycle
Thioredoxin-dependent peroxide reductase (Prdx3)	5	cysteine-type endopeptidase inhibitor activity involved in apoptotic process
Heat shock cognate 71 kDa protein (Hspa8)	5	protein quality control system (folding proteins and degredation), co-chaperone, signaling receptor binding and ubiquitin protein ligase binding
F-actin-capping protein alpha-2 (Capza2)	4	actin filament binding
Calumenin (Calu)	4	calcium ion binding and enzyme inhibitor activity
Heat shock factor-binding protein 1 (Hsbp1)	3	transcription corepressor activity
Granulins (Grn)	3	chaperone binding, cytokine activity, growth factor activity and RNA binding
Reticulocalbin-2 (Rcn2)	3	calcium ion binding
Elongation factor 1-beta (Eef1b)	3	guanyl-nucleotide exchange factor activity and translation elongation factor activity
14-3-3 protein epsilon (Ywhae)	3	recognition of a phosphoserine or phosphothreonine motif
Hemoglobin beta-1 (Hbb-b1)	3	oxygen carrier activity
Nuclease-sensitive element-binding protein 1 (Ybx1)	3	DNA-binding transcription activator, transcription factor binding
Elongation factor 1-delta (Eef1d)	3	activating transcription factor bindingand translation elongation factor activity
Dynein light chain 1, cytoplasmic (Dynll1)	2	motor activity and scaffold protein binding
Myosin light polypeptide 6 (Myl6)	2	motor activity and actin-dependent ATPase activity
Barrier-to-autointegration factor (Banf1)	2	DNA binding and enzyme binding
Dynein light chain 2, cytoplasmic (Dynll2)	2	motor activity and scaffold protein binding
60S acidic ribosomal protein P2 (Rplp2)	2	structural constituent of ribosome
Complement component 1 Q (C1qbp)	2	complement component C1q binding, mRNA binding and translation activator
Inter-alpha-trypsin inhibitor heavy chain H3 (Itih3)	2	binding protein between hyaluronan and other matrix protein
Reticulon-4 (Rtn4)	2	cadherin binding and neurite growth regulatory factor
Ig kappa chain V-III region PC 2413 (Kv3a5)	1	adaptive immune response
Ataxin-10 (Atxn10)	1	neuritogenesis
Protein LSM12 homolog (Lsm12)	1	interactor/competitor of ATXN2
Eukaryotic translation initiation factor 3 H (Eif3h)	1	translation initiation factor activity
Annexin A2 (Anxa2)	1	calcium-dependent phospholipid and cytoskeletal protein binding
Calmodulin (Calm1)	1	calcium-dependent protein and protein kinase binding
FAST kinase domain-containing protein 2 (Fastkd2)	1	protein kinase activity
Haptoglobin (Hp)	1	hemoglobin binding
Hippocalcin-like protein 1 (Hpcal1)	1	calcium ion binding
Ras-related protein Rab-33A (Rab33a)	1	GTPase activity
Ubiquitin-protein ligase E3B (Ube3b)	1	ubiquitin conjugating enzyme activity

Proteins identified by LC-MS/MS in the NCAM2-immunoprecipitated cerebral cortex of postnatal mice samples obtained with a False Discovery Rate ≤ 0.01% and detected with two or more peptides. The table shows the proteins, the number of different peptides (Ptd) found from each protein and the function of the proteins.

**Table 2:** Gene Ontology (GO) terms enriched for Biological Process, Molecular Function and Cellular Components in the NCAM2 interactors.

GOs Enrichment for Biological Process		
GO	Term Description	Proteins
GO:0030030	cell projection organization	Actb,Atxn10,Camk2a,Camk2b,Capzb,Dynll1,Dynll2,Map1b, Map2,Nefl,Nefm,Rtn4,Tubb4a
GO:0007010	cytoskeleton organization	Actb,Actc1,Capzb,Ina,Map1b,Map2,Nefl,Nefm,Tuba1a, Tub1c,Tubb4a,Tubb6
GO:0007017	microtubule-based process	Dynll1,Dynll2,Map1b,Map2,Nefl,Nefm,Tuba1a,Tuba1c, Tubb4a,Tubb6
GO:0060052	neurofilament cytoskeleton organization	Ina,Nefl,Nefm
GO:0010769	regulation of cell morphogenesis involved in differentiation	C1qbp,Camk2b,Map1b,Map2,Nefl,Nefm,Rtn4
GO:0007399	nervous system development	Actb,Atxn10,Camk2a,Camk2b,Camk2d,Camk2g,Capzb,Grn,Ina,Map1b,Map2,Nefl,Nefm,Rtn4,Ywhae,Ywhag
GO:0010970	transport along microtubule	Dynll1,Dynll2,Map1b,Nefl,Nefm
GO:0031175	neuron projection development	Actb,Atxn10,Camk2a,Capzb,Map1b,Map2,Nefl,Nefm,Rtn4
GO:0048699	generation of neurons	Actb,Atxn10,Camk2a,Camk2b,Capzb,Grn,Map1b,Map2, Nefl, Nefm,Rtn4,Ywhae,Ywhag
GO:0006414	translational elongation	Eef1b2,Eef1d,Rplp2
GO:0050770	regulation of axonogenesis	Map1b,Map2,Nefl,Nefm,Rtn4
GO:0061564	axon development	Actb,Map1b,Map2,Nefl,Nefm,Rtn4
GO:0045664	regulation of neuron differentiation	Camk2b,Grn,Map1b,Map2,Nefl,Nefm,Rtn4,Ywhag
GO:0010975	regulation of neuron projection development	Camk2b,Grn,Map1b,Map2,Nefl,Nefm,Rtn4
**GOs enrichment for Molecular Function**		
GO	Term Description	Proteins
GO:0005200	structural constituent of cytoskeleton	Actb,Nefl,Tuba1a,Tuba1c,Tubb4a,Tubb6
GO:0005198	structural molecule activity	Actb,Ina,Myl6,Nefl,Nefm,Rplp2,Tuba1a,Tuba1c,Tubb4a, Tubb6
GO:0004683	calmodulin-dependent protein kinase activity	Camk2a,Camk2b,Camk2d,Camk2g
GO:0008092	cytoskeletal protein binding	Actb,Actc1,Anxa2,Camk2d,Capza2,Capzb,Dynll1,Dynll2, Map1b,Map2,Ywhag
GO:0005519	cytoskeletal regulatory protein binding	Map1b,Map2
GO:0044325	ion channel binding	Calm1,Camk2d,Ywhae,Ywhaz
GO:0003924	GTPase activity	Rab33a,Tuba1a,Tuba1c,Tubb4a,Tubb6
GO:0005509	calcium ion binding	Anxa2,Calm1,Calu,Hpcal1,Myl6,Rcn2,Tubb4a
GO:0097110	scaffold protein binding	Dynll1,Dynll2,Ywhae
GO:0003746	translation elongation factor activity	Eef1b2,Eef1d
**GOs enrichment for Cellular Component**		
GO	Term Description	Proteins
GO:0005856	cytoskeleton	Actb,Actbl2,Actc1,Calm1,Camk2b,Capza2,Capzb,Dynll1, Dynll2,Hsbp1,Hspa8,Ina,Map1b,Map2,Myl6,Nefl,Nefm, Tuba1a,Tuba1c,Tubb4a,Tubb6,Ywhae
GO:0099513	polymeric cytoskeletal fiber	Actc1,Capzb,Dynll1,Dynll2,Hspa8,Ina,Map1b,Map2,Nefl, Nefm, Tuba1a,Tuba1c,Tubb4a,Tubb6
GO:0014069	postsynaptic density	Actb,Camk2a,Camk2b,Camk2g,Hspa8,Map1b,Map2,Nefm, Rtn4,Ywhaz
GO:0043005	neuron projection	Actb,Atxn10,Calm1,Camk2a,Camk2b,Camk2d,Camk2g, Capzb,Dynll1,Hspa8,Map1b,Map2,Nefl,Nefm,Rtn4,Tubb4a,Ybx1,Ywhae
GO:0030424	axon	Actb,Calm1,Camk2a,Camk2d,Dynll1,Hspa8,Map1b,Map2, Nefl,Nefm,Rtn4,Tubb4a,Ywhae
GO:0098794	postsynapse	Actb,Camk2a,Camk2b,Camk2g,Capzb,Hspa8,Map1b,Map2,Nefm,Rtn4,Ywhaz
GO:0005874	microtubule	Dynll1,Dynll2,Hspa8,Map1b,Map2,Tuba1a,Tuba1c,Tubb4a,Tubb6
GO:0045202	synapse	Actb,Camk2a,Camk2b,Camk2d,Camk2g,Capzb,Grn,Hspa8,Map1b,Map2,Nefm,Rtn4,Ywhaz
GO:0005883	neurofilament	Ina,Nefl,Nefm
GO:0015630	microtubule cytoskeleton	Calm1,Camk2b,Dynll1,Dynll2,Hspa8,Map1b,Map2,Tuba1a, Tuba1c,Tubb4a,Tubb6,Ywhae
GO:0030426	growth cone	Calm1,Map1b,Map2,Nefl,Rtn4,Ywhae
GO:0016529	sarcoplasmic reticulum	Calu,Camk2b,Camk2d,Camk2g
GO:0030425	dendrite	Atxn10,Camk2a,Camk2b,Capzb,Hspa8,Map1b,Map2,Rtn4, Ybx1
GO:0150034	distal axon	Calm1,Hspa8,Map1b,Map2,Nefl,Rtn4,Ywhae
GO:0015629	actin cytoskeleton	Actb,Actc1,Capza2,Capzb,Dynll2,Map2,Myl6
GO:0005853	eukaryotic translation elongation factor 1 complex	Eef1b2,Eef1d
GO:0099524	postsynaptic cytosol	Camk2a,Hspa8
GO:0005882	intermediate filament	Hspa8,Ina,Nefl,Nefm
GO:0008290	F-actin capping protein complex	Capza2,Capzb
GO:0044294	dendritic growth cone	Map2,Rtn4
GO:0043194	axon initial segment	Camk2d,Map2

List of the most relevant GOs involved in brain development, neuronal differentiation and synaptic formation. GOs enriched for Biological Process, Molecular Function and Cellular Components in NCAM2 interactome. The table shows the GO term identifier, the term description and the list of proteins detected in the mass spectrometry for each specific GO term.

**Table 3:** Kyoto Encyclopedia of Genes and Genomes (KEGGS) and The Reactome pathways enriched in NCAM2 interactome.

KEGGs Enrichment		
KEGG	Term Description	Proteins
mmu04722	Neurotrophin signaling pathway	Calm1,Camk2a,Camk2b,Camk2d,Camk2g,Ywhae
mmu04720	Long-term potentiation	Calm1,Camk2a,Camk2b,Camk2d,Camk2g
mmu04012	ErbB signaling pathway	Camk2a,Camk2b,Camk2d,Camk2g
mmu04540	Gap junction	Tuba1a,Tuba1c,Tubb4a,Tubb6
mmu04020	Calcium signaling pathway	Calm1,Camk2a,Camk2b,Camk2d,Camk2g
mmu04024	cAMP signaling pathway	Calm1,Camk2a,Camk2b,Camk2d,Camk2g
mmu04360	Axon guidance	Camk2a,Camk2b,Camk2d,Camk2g
**Reactome pathways enriched**		
Reactome	Term Description	Proteins
MMU-195258	RHO GTPase Effectors	Actb,Calm1,Dynll1,Dynll2,Myl6,Tuba1a,Tuba1c,Tubb4a, Tubb6,Ywhae,Ywhag,Ywhaz
MMU-442729	CREB phosphorylation through the activation of CaMKII	Calm1,Camk2a,Camk2b,Camk2d,Camk2g,Nefl
MMU-442982	Ras activation upon Ca2+ influx through NMDA receptor	Calm1,Camk2a,Camk2b,Camk2d,Camk2g,Nefl
MMU-199991	Membrane Trafficking	Actb,Capza2,Capzb,Dynll1,Dynll2,Rab33a,Tuba1a,Tuba1c, Tubb4a,Tubb6,Ywhae,Ywhag,Ywhaz
MMU-438066	Unblocking of NMDA receptors, glutamate binding and activation	Camk2a,Camk2b,Camk2d,Camk2g,Nefl
MMU-5576892	Phase 0-rapid depolarization	Calm1,Camk2a,Camk2b,Camk2d,Camk2g
MMU-190828	Gap junction trafficking	Actb,Tuba1a,Tuba1c,Tubb4a,Tubb6
MMU-190840	Microtubule-dependent trafficking of connexons from Golgi to the plasma membrane	Tuba1a,Tuba1c,Tubb4a,Tubb6
MMU-399719	Trafficking of AMPA receptors	Camk2a,Camk2b,Camk2d,Camk2g
MMU-5673001	RAF/MAP kinase cascade	Actb,Calm1,Camk2a,Camk2b,Camk2d,Camk2g,Nefl
MMU-1640170	Cell Cycle	Dynll1,Dynll2,Tuba1a,Tuba1c,Tubb4a,Tubb6,Ywhae, Ywhag,Ywhaz
MMU-5620912	Anchoring of the basal body to the plasma membrane	Dynll1,Tuba1a,Tubb4a,Ywhae,Ywhag

List of the most relevant KEGGs and Reactome pathways enriched in NCAM2 interactome. The table presents the KEGGs or the Reactome pathways identifier, the term description and the list of proteins detected in the mass spectrometry for each specific item.

## Supplementary Materials

The following are available online at https://www.mdpi.com/article/10.3390/ijms22147404/s1.
