# Supplementary material for: New Partners Identified by Mass Spectrometry Assay Reveal Functions of NCAM2 in Neural Cytoskeleton Organization
Source: Int J Mol Sci. 2021 Jul 9;22(14):7404. doi: 10.3390/ijms22147404 (PMC8304497; doi:10.3390/ijms22147404)
Supplement: Supplementary file 1 [file ijms-22-07404-s001.zip › Supplementary Materials.pdf]

Supplementary Materials

Figure S1: NCAM2 interacts with Actin. (**A**) NCAM2.1 and Actin interaction detection by Western-blot after co-immunoprecipitation experiments from P10-P15 mouse cortex and hippocampal protein extracts. Actin was detected after NCAM2.1 immunoprecipitation. (**B**) Quantification of Actin signal detected in WBs from (A). **** $p \leq 0.0001$; one-way ANOVA using Tukey comparison post-test.

Table S1: NCAM2 interactome. Proteins identified by LC-MS/MS in the NCAM2 immunoprecipitated samples obtained with False Discovery Rate ≤ 0.01%. The table shows the proteins, the number of different peptides (Ptd) and the coverage of these proteins.

Table S2: Mass spectrometry results. Proteins identified by LC-MS/MS in the NCAM2 immunoprecipitated samples obtained with False Discovery Rate ≤ 0.01% and detected with two or more peptides. The table shows the proteins detected with different peptides (Ppts) in the different experiments and with different antibodies. Proteins in red were detected using different antibodies (against extracellular domain of NCAM2 detecting both isoforms or against the intracellular domain of NCAM2.1); * marks proteins found in two different and independent experiments. Seven proteins were detected with both criteria.

Table S3: Gene Ontology (GO) terms enriched for Biological Process in the NCAM2 interactors. The table shows the GO term identifier, the term description, the number of genes observed, the background gene camp, the False Discovery Rate, the list of the matching proteins in the network (IDs) and the list of the matching proteins in the network (labels).

Table S4: Gene Ontology (GO) terms enriched in Molecular Function in the NCAM2's interactors. The table shows the GO term identifier, the term description, the number of genes observed, the background gene camp, the False Discovery Rate, the list of the matching proteins in the network (IDs) and the list of the matching proteins in the network (labels).

Table S5: Gene Ontology (GO) terms enriched in Cellular Components. The table shows the GO term identifier, the term description, the number of genes observed, the background gene camp, the False Discovery Rate, the list of the matching proteins in the network (IDs) and the list of the matching proteins in the network (labels).

Table S6: The Kyoto Encyclopedia of Genes and Genomes (KEGGS) pathways terms enriched. The table presents the KEGGs identifier, the term description, the number of genes observed, the background gene camp, the False Discovery Rate, the list of the matching proteins in the network (IDs) and the list of the matching proteins in the network (labels).

Table S7: The Reactome pathways enriched. The table presents the Reactome pathways identifier, the term description, the number of genes observed, the background gene camp, the False Discovery Rate, the list of the matching proteins in the network (IDs) and the list of the matching proteins in the network (labels).

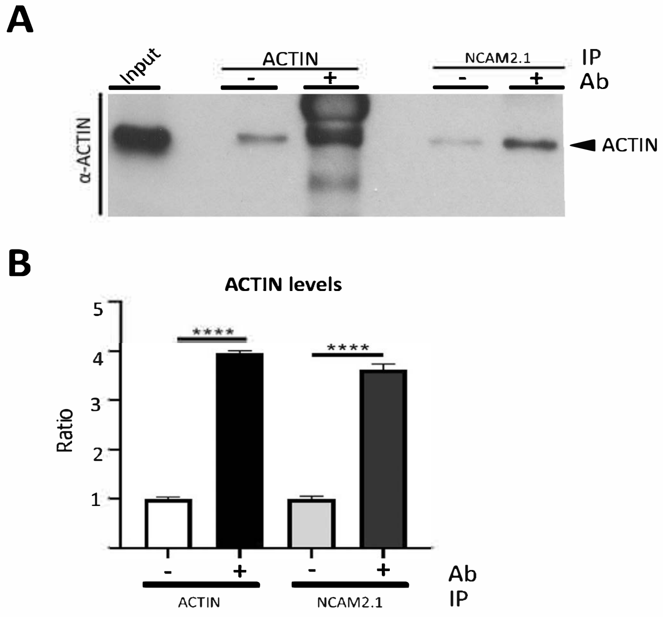

Supplementary Figure S1

Protein (gene)	Ptd.	Coverage
Microtubule-associated protein 2 (Map2)	24	21,50
Neurofilament light polypeptide (Nefl)	20	42,36
Actin, cytoplasmic 1 (Actb)	19	67,47
Neurofilament medium polypeptide (Nefm)	19	30,31
Tubulin beta-4A (Tubb4a)	18	60,59
Alpha-internexin (Ina)	17	37,33
Tubulin alpha-1A (Tuba1a)	13	44,79
Tubulin alpha-1C (Tuba1c)	12	39,64
Actin, alpha cardiac muscle 1 (Actc1)	11	31,56
Calcium/calmodulin-dependent protein kinase type II beta (Camk2b)	11	28,97
Calcium/calmodulin-dependent protein kinase type II alpha(Camk2a)	10	29,71
Microtubule-associated protein 1B (Map1b)	9	6,86
Beta-actin-like protein 2 (Actbl2)	8	27,39
F-actin-capping protein beta (Capzb)	8	45,49
14-3-3 protein zeta/delta (Ywhaz)	8	37,96
Tubulin beta-6 chain (Tubb6)	7	19,24
Calcium/calmodulin-dependent protein kinase type II delta (Camk2d)	6	16,43
Calcium/calmodulin-dependent protein kinase type II gamma (Camk2g)	6	13,61
14-3-3 protein gamma (Ywhag)	6	32,39
Enhancer of rudimentary homolog (Erh)	5	43,27
Thioredoxin-dependent peroxide reductase (Prdx3)	5	29,18
Heat shock cognate 71 kDa protein (Hspa8)	5	11,15
F-actin-capping protein alpha-2 (Capza2)	4	20,63
Calumenin (Calu)	4	23,17
Heat shock factor-binding protein 1 (Hsbp1)	3	72,37
Granulins (Grn)	3	7,13
Reticulocalbin-2 (Rcn2)	3	15,94
Elongation factor 1-beta (Eef1b)	3	28,89
14-3-3 protein epsilon (Ywhae)	3	14,51
Hemoglobin beta-1 (Hbb-b1)	3	23,81
Nuclease-sensitive element-binding protein 1 (Ybx1)	3	17,70
UPF0568 protein C14orf166 homolog (Cn166)	3	15,98
Elongation factor 1-delta (Eef1d)	3	17,08
Dynein light chain 1, cytoplasmic (Dynll1)	2	37,08
Myosin light polypeptide 6 (Myl6)	2	17,88
Barrier-to-autointegration factor (Banf1)	2	40,45
Dynein light chain 2, cytoplasmic (Dynll2)	2	20,22
60S acidic ribosomal protein P2 (Rplp2)	2	26,96
Complement component 1 Q (C1qbp)	2	15,83
Inter-alpha-trypsin inhibitor heavy chain H3 (Itih3)	2	4,39
Reticulon-4 (Rtn4)	2	2,47
Ig kappa chain V-III region PC 2413 (Kv3a5)	1	14,41
Ataxin-10 (Atxn10)	1	3,16
Protein LSM12 homolog (Lsm12)	1	6,67
Eukaryotic translation initiation factor 3H (Eif3h)	1	5,40
Annexin A2 (Anxa2)	1	2,95
Calmodulin (Calm1)	1	11,41
FAST kinase domain-containing protein 2 (Fastkd2)	1	2,76
Haptoglobin (Hp)	1	3,17
Hippocalcin-like protein 1 (Hpcal1)	1	10,36
Ras-related protein Rab-33A (Rab33a)	1	8,44
Ubiquitin-protein ligase E3B (Ube3b)	1	0,84

Protein (gene)	Ptd.	Coverage
60S acidic ribosomal protein P1 (Rplp1)	1	14,04
Adenomatous polyposis coli protein 2 (Apc2)	1	1,67
BRCA1-associated RING domain 1 (Bard1)	1	2,35
Carnitine O-palmitoyltransferase 1 (Cpt1a)	1	2,72
Cell cycle exit and neuronal differentiation protein 1 (Cend1)	1	10,74
Coiled-coil domain-containing 117 (Ccdc117)	1	13,72
Coiled-coil domain-containing 39 (Ccdc39)	1	1,49
Coiled-coil domain-containing 84 (Ccdc84)	1	1,81
Collagen alpha-1(XIV) chain (Col14a1)	1	0,45
Collectin-11 (Colec11)	1	15,44
Cytochrome c oxidase subunit NDUFA4 (Ndufa4)	1	14,63
Desmoglein-1-alpha (Dsg1a)	1	1,23
DNA helicase INO80 (Ino80)	1	0,51
Eukaryotic translation initiation factor 3F (Eif3f)	1	5,26
Exportin-6 (Xpo6)	1	1,78
Ferritin heavy chain (Fth1)	1	3,85
Galactoside 2-alpha-L-fucosyltransferase 1 (Fut1)	1	10,11
Galactosylgalactosylxylosylprotein 3-beta-glucuronosyltransferase 2 (B3gat2)	1	1,85
Galectin-9 (Lgals9)	1	4,53
Glyceraldehyde-3-phosphate dehydrogenase (Gapdh)	1	4,20
GRB2-associated-binding protein 2 (Gab2)	1	2,56
Lysozyme C-1 (Lyz1)	1	8,11
Matrilin-2 (Matn2)	1	4,08
MPN domain-containing protein (Mpnd)	1	2,87
N-acylglucosamine 2-epimerase (Renbp)	1	1,40
Non-lysosomal glucosylceramidase (Gba2)	1	4,25
NudC domain-containing protein 2 (Nudcd2)	1	12,74
PAX-interacting protein 1 (Paxip1)	1	0,95
Protein broad-minded (Tbc1d32)	1	0,62
Pyruvate dehydrogenase E1 component subunit beta, mitochondrial (Pdhb)	1	4,46
Rab GTPase-activating protein 1 (Rabgap1)	1	1,79
Rootletin (Crocc)	1	0,50
Serine protease 28 (Prss28)	1	3,28
Signal recognition particle subunit SRP68 (Srp68)	1	1,28
Single-stranded DNA-binding protein, (Ssbp1)	1	9,87
Solute carrier family 7 member 13 (Slc7a13)	1	2,72
Stimulated by retinoic acid gene 8 (Stra8)	1	4,33
Sushi domain-containing protein 2 (Susd2)	1	5,12
Synaptotagmin-like protein 5 (Sytl5)	1	1,46
Thrombospondin type-1 domain-containing protein 7B (Thsd7b)	1	1,12
Transferrin receptor protein 1 (Tfrc)	1	2,88
Transmembrane protease serine 5 (Tmprss5)	1	3,30
Tripartite motif-containing protein 75 (Trim75)	1	2,78
Tumor protein D52 (Tpd52)	1	5,36
Tyrosine-protein kinase JAK1 (Jak1)	1	0,52
Unconventional myosin-Ib (Myo1b)	1	2,62
UPF0577 protein KIAA1324 (Kiaa1324)	1	4,16
UPF0704 protein C6orf165 homolog	1	3,86

Protein	Total Ppts	Experiment 1		Experiment 2	
		NCAM2 Ppts	NCAM2.1 Ppts	NCAM2 Ppts	NCAM2.1 Ppts
Microtubule-associated protein 2 (Map2) *	24		4		22
Neurofilament light polypeptide (Nefl) *	20		6		20
Actin, cytoplasmic 1 (Actb) *	19	1	9	2	17
Neurofilament medium polypeptide (Nefm) *	19		3		19
Tubulin beta-4A (Tubb4a)	18				18
Alpha-internexin (Ina) *	17		1		17
Tubulin alpha-1A (Tuba1a) *	13		3	1	13
Tubulin alpha-1C (Tuba1c)	12		3	1	
Actin, alpha cardiac muscle 1 (Actc1) *	11	1	5		10
Calcium/calmodulin-dependent protein kinase type II beta (Camk2b)	11				11
Calcium/calmodulin-dependent protein kinase type II alpha(Camk2a) *	10		1		10
Microtubule-associated protein 1B (Map1b)	9				9
Beta-actin-like protein 2 (Actbl2)	8				7
F-actin-capping protein beta (Capzb)	8				8
14-3-3 protein zeta/delta (Ywhaz)	8			1	8
Tubulin beta-6 chain (Tubb6)	7	1			
Calcium/calmodulin-dependent protein kinase type II delta (Camk2d)	6				6
Calcium/calmodulin-dependent protein kinase type II gamma (Camk2g)	6				6
14-3-3 protein gamma (Ywhag)	6			1	6
Enhancer of rudimentary homolog (Erh) *	5		2		5
Thioredoxin-dependent peroxide reductase (Prdx3) *	5		3		4
Heat shock cognate 71 kDa protein (Hspa8) *	5	1	4	1	2
F-actin-capping protein alpha-2 (Capza2)	4				4
Calumenin (Calu) *	4		1		3
Heat shock factor-binding protein 1 (Hsbp1) *	3	1		3	3
Granulins (Grn) *	3	3	2	3	1
Reticulocalbin-2 (Rcn2)	3			3	2
Elongation factor 1-beta (Eef1b) *	3		2		3
14-3-3 protein epsilon (Ywhae)	3			1	3
Hemoglobin beta-1 (Hbb-b1)	3				3
Nuclease-sensitive element-binding protein 1 (Ybx1)	3				3
UPF0568 protein C14orf166 homolog (Cn166)	3				3
Elongation factor 1-delta (Eef1d) *	3		1		2
Dynein light chain 1, cytoplasmic (Dynll1) *	2		1	1	2
Myosin light polypeptide 6 (Myl6)	2				2
Barrier-to-autointegration factor (Banf1)	2				2
Dynein light chain 2, cytoplasmic (Dynll2)	2		2	1	
60S acidic ribosomal protein P2 (Rplp2) *	2		1		2
Complement component 1 Q subcomponent-binding protein, mitochondrial (C1qbp)	2		2		
Inter-alpha-trypsin inhibitor heavy chain H3 (Itih3)	2				2
Reticulon-4 (Rtn4)	2				2
Ig kappa chain V-III region PC 2413 (Kv3a5)	1			1	
Ataxin-10 (Atxn10) *	1		1		1
Protein LSM12 homolog (Lsm12) *	1		1		1
Eukaryotic translation initiation factor 3 subunit H (Eif3h)	1				1
Annexin A2 (Anxa2)	1	1	1		
Calmodulin (Calm1)	1	1			1
FAST kinase domain-containing protein 2 (Fastkd2)	1				1
Haptoglobin (Hp)	1				1
Hippocalcin-like protein 1 (Hpcal1)	1			1	1
Ras-related protein Rab-33A (Rab33a)	1			1	1
Ubiquitin-protein ligase E3B (Ube3b)	1				1

SUPPLEMENTARY TABLE S2
